# Epigenetic aging of the demographically non-aging naked mole-rat

**DOI:** 10.1038/s41467-022-27959-9

**Published:** 2022-01-17

**Authors:** Csaba Kerepesi, Margarita V. Meer, Julia Ablaeva, Vince G. Amoroso, Sang-Goo Lee, Bohan Zhang, Maxim V. Gerashchenko, Alexandre Trapp, Sun Hee Yim, Ake T. Lu, Morgan E. Levine, Andrei Seluanov, Steve Horvath, Thomas J. Park, Vera Gorbunova, Vadim N. Gladyshev

**Affiliations:** 1 Division of Genetics, Department of Medicine, Brigham and Women’s Hospital and Harvard Medical School, Boston, MA 02115 USA; 2 Department of Pathology, Yale School of Medicine, New Haven, CT 06510 USA; 3 Departments of Biology and Medicine, University of Rochester, Rochester, NY 14627 USA; 4 Laboratory of Integrative Neuroscience, Department of Biological Sciences, University of Illinois at Chicago, Chicago, IL 60607 USA; 5 Department of Human Genetics, David Geffen School of Medicine, University of California, Los Angeles, CA 90095 USA; 6 Department of Biostatistics, Fielding School of Public Health, University of California, Los Angeles, CA 90095 USA

**Keywords:** Epigenetics, Ageing, Genome informatics

## Abstract

The naked mole-rat (NMR) is an exceptionally long-lived rodent that shows no increase of mortality with age, defining it as a demographically non-aging mammal. Here, we perform bisulfite sequencing of the blood of > 100 NMRs, assessing > 3 million common CpG sites. Unsupervised clustering based on sites whose methylation correlates with age reveals an age-related methylome remodeling, and we also observe a methylome information loss, suggesting that NMRs age. We develop an epigenetic aging clock that accurately predicts the NMR age. We show that these animals age much slower than mice and much faster than humans, consistent with their known maximum lifespans. Interestingly, patterns of age-related changes of clock sites in *Tert* and *Prpf19* differ between NMRs and mice, but there are also sites conserved between the two species. Together, the data indicate that NMRs, like other mammals, epigenetically age even in the absence of demographic aging of this species.

## Abstract (web summary)

The exceptionally long-lived naked mole-rat is characterized by the lack of increased mortality with aging. Here the authors perform epigenetic studies to show that naked mole-rats epigenetically age despite their non-increasing mortality rate.

## Introduction

The naked mole-rat (*Heterocephalus glaber*, NMR) is the longest-lived rodent with a maximum lifespan of more than 30 years in captivity^1–3^. In contrast, the maximum lifespan of a similarly sized rodent, the house mouse (*Mus musculus*), is 4 years in the laboratory^4,5^. The NMR lifespan is five-fold greater than that predicted allometrically based on its body size^2^. This extreme longevity of NMRs is accompanied by a non-increasing mortality rate that led to the conclusion that the NMR is a non-aging mammal^6–8^. Although the lack of demographic aging is most confidently revealed up to the age of 12 years (because the vast majority of examined animals were within this age range)^6,9^, this age span is two times longer than the lifespan expected based on body size and 20-fold longer than the possible onset of reproduction^7^. The NMR was shown to resist age-related diseases such as cancer and cardiovascular disease^10–12^, shows no change in body composition and metabolic profiles with age^13^, and in general, its observed health deterioration (e.g., cataracts and osteoarthritis) do not appear to change in severity or frequency as age increases^8^. However, NMRs do exhibit age-related changes, such as decreased activity and altered (lighter and parchment-like) skin^1,2,5^. Furthermore, proteomic and transcriptomic analyses of NMR livers revealed progressive age-dependent changes at the molecular level^14^. How these physiological age-related changes relate to the absence of demographic aging (i.e., non-increasing mortality rate with age) is unclear, but it was discussed that the mortality rate is not always the best representation of aging^15^.

Epigenetic changes, which lead to dysregulation of transcriptional and chromatin networks, may be crucial components of the aging process^16^. Human age can be accurately predicted using the methylation levels of a few hundred CpG sites in the blood, and age quantification is possible even across tissues^17,18^. Epigenetic clocks may measure various aspects of human aging as they show age acceleration linked to age-related conditions such as mortality, impaired cognitive performance, frailty, Parkinson’s disease and Werner syndrome^19^. Similarly, several mouse clocks have been developed, including our blood and multi-tissue clocks covering the entire mouse lifespan and showing a reduced biological age in response to longevity interventions (e.g. calorie restriction and growth hormone receptor knockout)^20–25^. Both mouse and human clocks also showed a dramatic decrease in epigenetic age during reprogramming of adult fibroblasts to induced pluripotent stem cells^18,20,21,25,26^. The epigenome of other mammals also steadily changes with age allowing to build species-specific and even pan-species clocks^27–29^.

In the current work, we developed an NMR epigenetic aging clock using a high-resolution methylome approach and examined age-related DNA methylation (DNAm) changes in this species against those in other mammals. Additionally, a recent study reported an NMR clock in the skin and liver using a targeted analysis of 51 pre-defined CpGs^30^. Also, array-based epigenetic aging clocks were developed, trained on 27,917 pre-defined CpG sites^31^. To thoroughly investigate the possibility of NMR aging, we subjected 107 NMR blood samples to bisulfite sequencing, which allowed the analysis of over 3 million common CpG sites. Using this resource, we were able to identify patterns of age-related DNA methylation changes in the NMR, demonstrating that these animals age epigenetically (i.e., exhibit age-associated epigenetic changes), although in a unique way compared to other mammals.

## Results

### Global changes in the NMR methylome with age

**Fig. 1:**
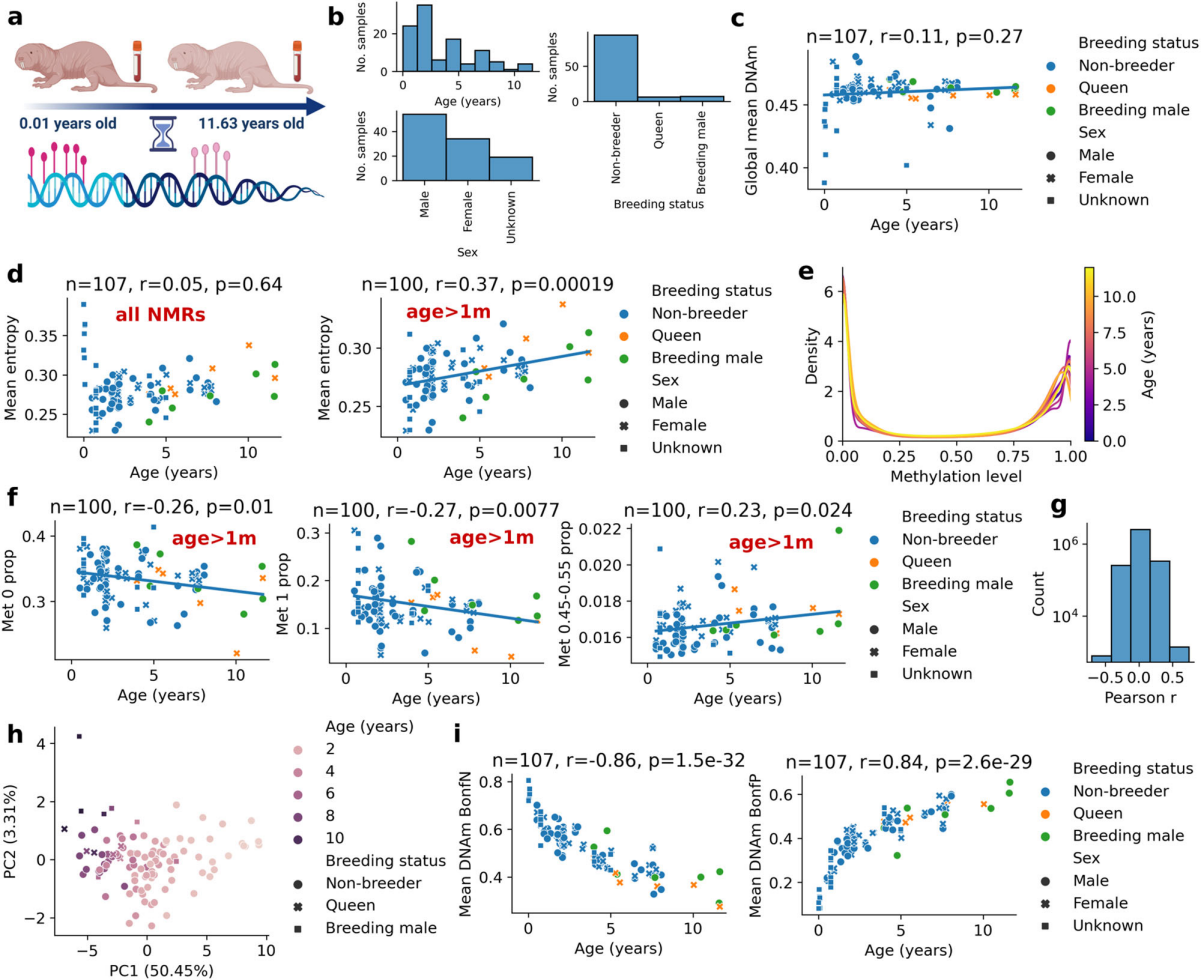
Global changes in the NMR methylome during aging. **a** Schematic of the study design suggesting changes of the NMR blood methylome during aging **b** Sample distribution of 107 NMR blood samples used in this study (one sample per animal). **c** Global DNAm level of all 3,089,098 CpG sites as a function of age. **d** Mean Shannon entropy of CpG levels (*Mean entropy*) for all NMRs (left panel) and NMRs older than 1 month (right panel). **e** Density plot of methylation levels for a representative NMR methylome in each year. **f** Proportion of methylation levels 0, 1 and ~0.5 for NMRs older than 1 month (*Met 0 prop*, *Met 1 prop*, *Met 0.45-0.55 prop,* respectively). **g** Histogram of Pearson correlation coefficients of methylation levels and ages for each CpG site in the NMR. **h** PCA of 758 CpG sites that are significant after Bonferroni correction **i** Mean methylation level of negatively correlated CpG sites (*Mean DNAm BonfN*) and positively correlated CpG sites (*Mean DNAm BonfP*). Number of samples (*n*), Pearson correlation coefficient (*r*), its two-sided *p*-value (*p*) are indicated for the specific panels. Source data are provided as a Source Data file.

To determine whether the DNA methylome of the NMR changes with age and characterize patterns of these changes, we obtained high-resolution DNA methylomes of 107 blood samples derived from both breeding and non-breeding NMRs ranging in age from 0.01 to 11.63 years (Fig. 1a, b, Supplementary Data 1). We adapted a blood collection procedure for NMRs to accommodate a naturally low blood pressure of the animals. The blood was collected from either the tail vein or ventral artery of animals using a procedure that did not require animal sacrifice (see Methods). The resulting samples were sequenced by reduced representation bisulfite sequencing (RRBS) at high coverage, such that we achieved over 3 million CpG sites common to all samples.

Global mean DNA methylation (i.e. average methylation level of 3,089,098 common CpGs) remained relatively stable with age in NMRs (mean: 0.4597, std: 0.014) and remained slightly demethylated (i.e. less than 0.5 for all samples) (Fig. 1c). In contrast, the methylome entropy (mean Shannon entropy of CpG methylation levels) showed a distinct U-shaped pattern: a sharp decrease at the youngest ages (< 1 month) followed by a gradual age-related increase during the whole lifespan (Fig. 1d, *p* = 0.00019). A density plot of methylation levels suggested that the age-related entropy change may be driven by the decrease in extreme values (i.e., methylation levels 0 or 1) and the slight increase of the values close to 0.5 (Fig. 1e). Indeed, the proportion of sites with methylation levels 0 and 1 significantly decreased with age and the methylation levels close to 0.5 (i.e., between 0.45 and 0.55) significantly increased for NMRs older than 1 month (Fig. 1f). Altogether, except for the youngest ages that correspond to development, we observed an information loss of the NMR methylome during aging (as measured by Shannon entropy), suggesting a possibility that NMRs age.

We also examined mean methylation levels of NMR gene promoters. Mean promoter methylation across all 17,823 NMR genes covered by our data significantly increased with age (Supplementary Fig. 1a). The correlation of mean promoter methylation and age ranged from −0.5518 to 0.7821 for the 320 genes that remained significant after Bonferroni correction (Supplementary Fig. 1b, Supplementary Data 2). *Tert* (telomerase reverse transcriptase) promoter showed the strongest correlation with age (*r* = 0.7821, *p* = 2.67e-23), and 8 other gene promoters showed correlation coefficients greater than 0.7 (Supplementary Fig. 1c).

To further investigate global changes in NMR methylation, we calculated Pearson correlation coefficients of methylation level and age for each CpG site. Approximately 9% (279,990 CpGs) of 3,089,098 CpG sites showed significant correlation with age (Fig. 1g). 758 CpG sites remained significant after Bonferroni correction (two-sided p-value below 1.62e-08). Principal component analysis (PCA) of 758 CpG sites suggested an age-related remodeling of the NMR methylome (Fig. 1h). The mean methylation level of both negatively correlated (*N* = 95) and positively correlated (*N* = 663) CpG sites highly correlated with age and showed a distinct pattern of decelerated aging (Fig. 1i). These results, together with previous findings^30,31^, suggest that an accurate epigenetic clock may be developed for the NMR by using RRBS.

### NMR blood aging clock based on high-resolution methylomes

**Fig. 2:**
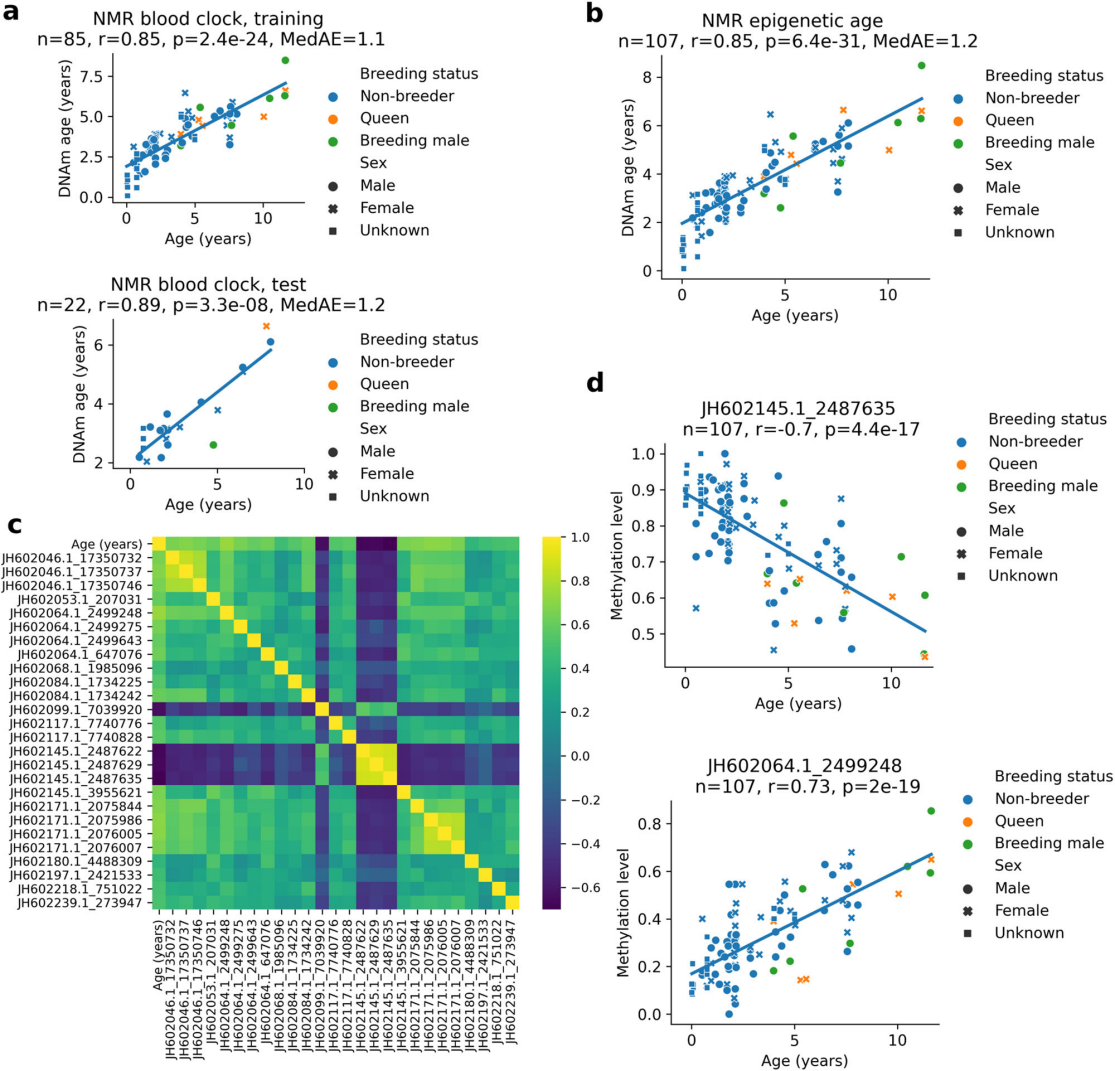
NMR blood aging clock based on high-resolution DNA methylomes. **a** Training (upper panel) and testing (lower panel) of the NMR blood clock. **b** Predicted age (*DNAm age*) of NMR samples by using ElasticNet regression via 5-fold cross-validation. Linear regression line of the predicted ages is also shown. **c** Pearson correlation coefficients among the age and the 26 CpG sites of the NMR blood clock. Names of clock sites include accession number of the genomic contig and the genomic position in the contig separated by ‘_’. **d** Age-related changes in methylation levels of clock CpG sites with the lowest and the highest correlations with age. Number of samples (*n*), Pearson correlation coefficient (*r*), its two-sided *p*-value (*p*) and the median absolute error (*MedAE*) are indicated for the specific panels. Source data are provided as a Source Data file.

To develop an NMR epigenetic clock, we used the 107 blood samples, selecting the CpG sites that were sufficiently covered (by at least 5 reads) in 100% of the samples generating a methylation profile data table containing 430,080 high-quality CpG sites. We trained an ElasticNet regression model using the random 80% of samples (*n* = 85) and tested on the remaining samples (*n* = 22) (Fig. 2a). The lambda parameter was optimized on the training set by 10-fold cross-validation. To achieve reliable (e.g., test) age predictions for all study samples, we performed a 5-fold cross-validation repeating the same procedure (Supplementary Fig. 2). The unified test predictions of cross validation showed high correlation with chronological age (*r* = 0.85, *p* = 6.4e-31), suggesting that the NMR blood displays epigenetic aging (Fig. 2b, Supplementary Data 3), similarly to what has been shown recently by using other methods^30,31^. While age acceleration did not deviate from zero when we considered all (young and old) queens, (*n* = 6, *p* = 0.9896), age acceleration of the two oldest queens (age >10 years) was lower compared to younger queens (*p* = 0.043). This finding is consistent with a recent report of slightly slower progression of aging in naked mole-rat queens, which was observed more prominently when older animals, that were in the queen status for a long time, were considered^31^. In contrast, age acceleration of breeding males did not deviate significantly from zero (*p* = 0.6) and the oldest animals (age >10 years) showed no significant age acceleration compared to the younger ones (*p* = 0.632). Non-breeding females and non-breeding males showed neither age acceleration nor deceleration (*p* = 0.276, *p* = 0.311).

The NMR blood clock is based on a linear model of 26 CpG sites (Supplementary Data 4). The predicted age is calculated as the weighted average of methylation levels of the 26 CpG sites (‘clock sites’) plus 3.133 years (the intercept). All clock sites correlated with age and one another, most prominently the neighboring CpGs (Fig. 2c). The most negatively correlated and positively correlated clock sites showed *r* = −0.7 (*p* = 4.4e-17) and *r* = 0.73 (*p* = 2e-19), respectively (Fig. 2d). All 26 clock sites showed significant changes in DNAm levels with age, with different patterns of age-related changes (Supplementary Fig. 3).

### Patterns of age-related DNA methylation changes of NMR clock-associated genes

To elucidate genetic associations of the NMR blood clock, we investigated the methylation dynamics of clock CpG sites during aging. Twenty of the 26 clock sites were in the NMR gene bodies (exons or introns) or promoter regions (Supplementary Fig. 3). Interestingly, methylation levels of all gene-associated clock CpG sites increased with age, while the remaining non-gene-associated clock CpG sites showed either decreasing or increasing DNAm levels with age.

**Fig. 3:**
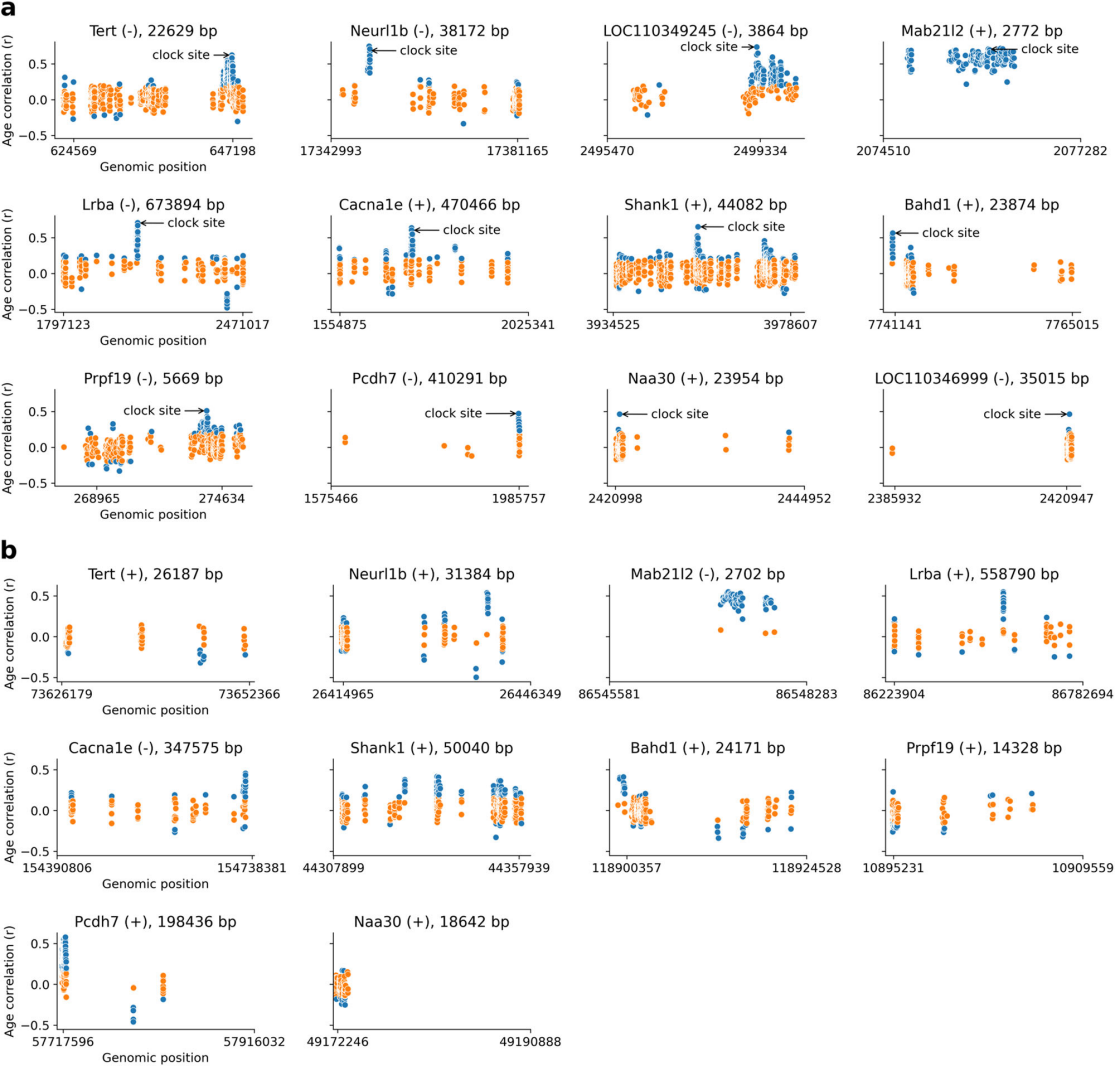
Patterns of age-related DNA methylation changes of NMR clock-associated genes. **a** Correlation coefficient with age (y-axis) for all covered CpG sites within an NMR clock-associated gene along its 1,500 bp long upstream or downstream flanking region including the promoter region (upstream). Gene name, strand and the length of the transcript are shown on the top. The start and end positions of genes are indicated on the x-axis. Transcription start site (TSS) shown in the right for the negative strand (-) genes and in the left for the positive strand (+) genes. Arrows point to the highest age-correlated clock CpG site for each gene. **b** Age-associated methylation patterns of mouse homologs of the NMR clock-associated genes for comparison. Significant correlations (p < 0.05) were colored blue. We calculated two-sided *p*-values without an adjustment for multiple comparison.

The RRBS dataset allowed us to investigate genes associated with clock sites at the base-pair level. We calculated correlation coefficients for all covered CpG sites within each gene along with 1,500 bp long upstream and downstream flanking regions including promoters (upstream). This procedure revealed 12 clock site-associated genes in the NMR: *Tert*, *Neurl1b*, *Mab21l2*, *LOC110349245*, *Lrba*, *Cacna1e*, *Shank1*, *Bahd1*, *Prpf19*, *Pcdh7*, *Naa30* and *LOC110346999* (Fig. 3a). We omitted *Cartpt* as a clock site-associated gene, as it had a low weight (0.02) in the NMR blood clock model and hence negligible influence on the predictions. In all cases, clock CpG sites showed one of the highest correlations with age compared to the other CpG sites in the gene. However, in most cases other highly correlated sites were in the neighborhood of NMR blood clock sites, suggesting that changes in methylation with age are not site-specific but rather region-specific.

We further found that methylation at most CpG sites within genes did not change significantly with age suggesting that only a distinct part of a gene is exposed to epigenetic aging. A notable exception was *Mab21l2*, for which all CpG sites showed a significant positive correlation with age.

### NMR clock-associated genes show similar aging patterns in the mouse except for *Tert* and *Prpf19*

We next tested whether the aging patterns of NMR clock-associated genes are similar to the aging patterns of their mouse orthologs. For this purpose, we utilized our previous mouse RRBS dataset (GSE80672). We used the methylation levels of whole blood samples from wild-type C57BL/6 mice (*n* = 153, age range: 0.67 m – 35 m) and analyzed 1,894,100 CpG sites that had sufficient read coverage (at least 5 reads) in at least 90% of the samples. Orthologs of 10 out of the 12 NMR clock-associated genes in the mouse genome could be identified (Fig. 3b).

We found that the age-related methylation pattern of *Tert* in the mouse was remarkably different from that of the NMR ortholog. In the NMR, the promoter region of *Tert* contained a large number of CpG sites with increasing methylation levels with age; instead, mouse *Tert* lacked significantly increasing CpG sites, including a cluster near the transcription start site (TSS). A similar behavior was observed for *Prpf19*, where CpG sites near the TSS were highly correlated with age in the NMR, but not in the mouse. Among other genes, *Neurl1b* contained multiple strongly age-correlated CpG sites in the gene body at approximately the same relative location as in the NMR homolog, and the same was observed for *Lrba*. *Mab21l2* in the mouse recapitulated a remarkable pattern observed in the NMR, wherein almost all its CpG sites significantly increased methylation levels with age (except several non-significant but still increasing cases). *Cacna1e* in both species contained one narrow cluster with highly age-correlated CpG sites but in a different relative location (near the TSS in the mouse while in the middle of the gene body in the NMR). *Shank1* showed multiple clusters of age-associated CpG sites, but the highest association was in the middle of the gene body in both species. *Bahd1* and *Pcdh7* contained a highly age-correlated cluster near the TSS in both species. *Naa30* showed significant correlation only in the promoter region in both species. This gene in the NMR contained a single outlier CpG site with high correlation, but it was missing in the mouse. Overall, the most striking differences between the mouse and NMR were seen in the case of *Tert* and *Prpf19*, while the remaining genes showed rather similar patterns. As *Tert* is an important aging-related gene and *Prpf19* is involved in DNA repair, it would be interesting to test whether these differences are linked to the extreme longevity and/or cancer resistance of the NMR.

To validate the results, we assessed another mouse blood RRBS dataset (GSE120132). We used the methylation levels of blood samples from female and male C57BL/6 (*n* = 50) and BALB/cByJ (*n* = 22) mice (age range: 1.65 – 21.28 months) and examined 1,074,105 CpG sites that had sufficient read coverage (at least 5 reads) in at least 90% of the samples. The methylation patterns of NMR clock-associated orthologs in the mouse genome were remarkably similar in this dataset compared to the first examined mouse dataset, further supporting our findings (Supplementary Fig. 4).

### Rates of age-related changes of NMR, mouse and human blood clock CpG sites

**Fig. 4:**
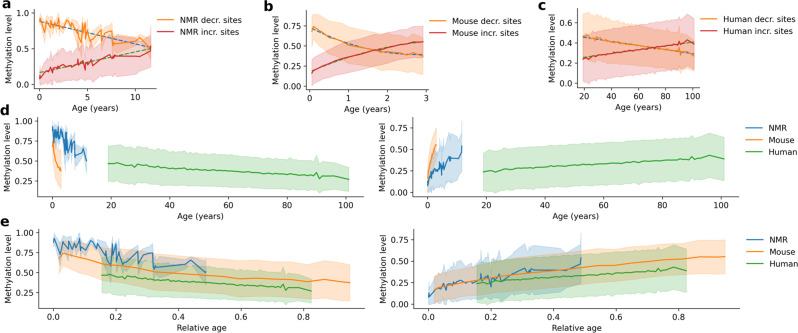
Comparing the rate of age-related changes in DNA methylation levels of NMR-, mouse- and human blood clock CpG sites. **a** Mean methylation level of CpG sites that decrease and increase methylation with age in the NMR blood clock. Linear regression and standard deviation are indicated by dashed lines and pale coloring around it. **b** Mean methylation level of CpG sites that decrease and increase methylation with age in the Petkovich et al. mouse blood clock^20^. Quadratic regression and standard deviation are indicated by dashed curves and pale coloring around it. **c** Mean methylation level of CpG sites that decrease and increase methylation with age in the Hannum human blood clock^17^. Linear regression and standard deviation are indicated by dashed lines and pale coloring around it. **d** Mean methylation level of decreasing CpG sites of the three clocks shown on the same age scale (left panel). The same is shown for increasing CpG sites (right panel). Standard deviation is indicated by pale coloring around the mean. **e** Mean methylation level of decreasing CpG sites of the three clocks shown on a relative age scale (age divided by the maximum lifespan of species) (left panel). The same is shown for increasing CpG sites (right panel). Standard deviation is indicated by pale coloring around the mean. Source data are provided as a Source Data file.

CpG sites of the NMR blood clock can be divided into two groups: sites whose methylation levels negatively correlate with age (*decreasing sites* hereafter) and sites whose methylation levels positively correlate with age (*increasing sites* hereafter). The decreasing sites tend to have a high methylation level at the age zero, while the increasing sites exhibit a low methylation level at that age. The same phenomenon was previously observed in mice and humans^17,20^. We assessed age-related changes in the average methylation level of the NMR clock CpG sites as it may be viewed as a proxy for the rate of aging (Fig. 4a). We calculated the same for the mouse by using the whole blood RRBS dataset (*n* = 153) and the corresponding blood clock^20^ (Fig. 4b). This clock contained 37 increasing and 53 decreasing CpG sites. Interestingly, decreasing mouse clock CpG sites started to decrease from a lower methylation level at the youngest age compared to the NMR sites, whereas the increasing mouse clock sites started to increase from a higher methylation level compared to the NMR sites. We also analyzed age-related changes of human blood clock CpG sites (*Hannum clock*) based on Illumina 450k methylation profiles of 656 blood samples from individuals with the age range from 19 to 101 years (GSE40279)^17^. This clock had 32 decreasing and 40 increasing sites. As in the NMR, we observed a linear trend of age-related changes in the human clock CpG sites for both decreasing and increasing CpG sites (Fig. 4c).

We then examined the three species on the same time scale, separately for decreasing and increasing sites (Fig. 4d). The exact difference in the aging rates between these species could not be calculated, as it changed differently with age in NMRs and humans on one side, and mice on the other. Moreover, our data covered mostly adult ages of the three species, and thus how these species aged during development could not be compared. It was clear, however, that NMRs epigenetically aged much slower than mice and much faster than humans (Fig. 4d), consistent with known maximum lifespans of the three species. The trend lines of the NMR exhibited a higher fluctuation compared to the other two species due to the lower number of clock CpG sites (especially in the case of decreasing sites, with *n* = 4). We also examined the NMR, mouse and human on the scale of relative age (age divided by the maximum lifespan; 31, 4, and 122.5 years, respectively, using the AnAge database^32^) (Fig. 4e). After this procedure, epigenetic changes during the whole lifespan showed similar relative age dynamics in the three species. We observed that the trend lines of decreasing and increasing sites crossed each other at 11.87, 1.66, and 70.69 years, respectively, for the NMR, mouse and human (Fig. 4a–c). It may indicate that a 11.87 year old NMR roughly correspondent to a 1.66 year old mouse or a 70.69-year-old human in terms of age-related epigenetic changes. The relative age of the cross-over was 0.383, 0.414, and 0.577, respectively, and it occurred at the methylation levels of 0.508, 0.447, and 0.352. Entropy is often linked to the aging process, and the methylation entropy is highest at 0.5 methylation. In line with this, the trend lines of decreasing and increasing sites converged around the 0.5 methylation level. Overall, multiple lines of evidence revealed clear epigenetic aging patterns in NMRs, which generally recapitulated the well-characterized aging patterns observed in mice and humans.

## Discussion

Considering that the NMR mortality rate does not change with age (most confidently, during the first 12 years of life), an important question is whether these animals age at all. We consider aging as an accumulation of deleterious age-related changes, which include molecular damage and other age-related negative consequences of metabolism and interaction with environment^33^. It is possible that an organism may age even in the period of life, when its mortality rate is constant or even when it decreases, as is the case of humans under the age of nine^15,34^; hence mortality rate does not always represent aging. Very recently, age-associated DNA methylation changes in the NMR skin and liver have been shown by a targeted analysis of 51 CpGs, with the top hit CpG showing a strong correlation (*r* = 0.88) with age^30^. In our study, we uncovered global changes in the NMR methylome during aging and developed an epigenetic aging clock for the NMR blood, demonstrating that NMRs do exhibit many age-related molecular alterations over their lifespan. Taken together with other recent studies examining other tissues^30,31^, these findings show that NMRs epigenetically age (exhibit age-related epigenetic changes) even if this process is not demographically observable (i.e., NMRs do not exhibit an increase in mortality rate with age). One may argue that the existence of epigenetic aging clocks for a species already means that these animals steadily change their epigenome with age, i.e., they age. In line with this argument, it was shown previously that mouse and human epigenetic aging clocks track the aging process and can estimate biological age^19,20^. This argument is based on the observed age acceleration in the case of age-related conditions in humans and evaluation of longevity interventions in mice. However, the NMR clock has not been evaluated in these settings, hence, in theory, it is possible, although unlikely, that age-related epigenetic changes in the NMR are not governed by damage accumulation but by some other processes, e.g., continued development. Application of human multi-tissue epigenetic clocks suggested that aging may be an unintended consequence of processes that are necessary for the development of the organism and tissue homeostasis thereafter; however the pace of aging may be affected by external factors^35^. Consistent with these ideas, we observed a steady increase in entropy during adult life of NMRs, again pointing to the possibility of NMR aging.

Several NMR genes that we found to contain epigenetic aging clock sites are linked to the hallmarks of aging^36^. One is telomerase, which protects chromosomes from telomere attrition^36^ and consists of two components: a reverse transcriptase catalytic subunit (*Tert*) and telomerase RNA (*Ter*), a template for telomere^37^. *Tert* shows a similar activity in NMRs and mice; however, NMRs have short telomeres, whereas in mice telomeres are extremely long^38^. We observed increased methylation of *Tert* promoter with age. Interestingly, *Tert* expression showed a stable level with age (e.g., when comparing newborn, 4-year-old and 20-year-old NMRs)^39^, and telomere length did not shorten, rather showed a mild elongation in the first 20 years of NMR life based on a qPCR approach^40^ and increased followed by a decrease based on another approach (qFISH)^41^.

Another gene, *Prpf19*, is involved in double-strand break (DSB) repair, which also offers a link to aging^42^. We recently reported that DSB repair is more efficient in long-lived rodents, including the NMR, compared to short-lived species such as mice^43^. An additional NMR blood clock site gene, *Bahd1*, supports heterochromatic gene silencing^44^, whereas *Naa30* maintains mitochondrial integrity^45^; both of these processes have been implicated in the aging process. Furthermore, *Neurl1B, Cana11e*, and *Pcdh7* are metal ion binding proteins that may be linked to aging, as metal ion dysregulation is a known component of aging^46^. While it is possible that increased methylation of *Tert* and *Prpf19* promoters contributes to NMR aging, we also found that the patterns of age-related changes of clock sites in these two genes differ between NMRs and mice. Whether this is related to drastically different lifespans of these two species or some other differences is unclear. Age-related human *Tert* promoter dynamics appears to be different from mice and NMRs as aging of oral epithelial cells was associated with hypomethylation of the human *Tert* promoter^47^. Furthermore, the local genome structure around the *Tert* gene in rodents is different from humans, implying they may have developed different strategies to regulate telomerase expression^48^.

An NMR clock site-associated gene, *Mab21l2*, showed a unique and remarkable pattern in both NMRs and mice: almost all covered CpG sites of this gene significantly increased with age. *Mab21l2* plays crucial roles in the retina and in ventral body wall formation in the mouse^49^. Interestingly, one of 29 clock sites of the recently developed epigenetic clock in the zebrafish is also located in the *Mab21l2* gene^29^. This clock site is in the promoter region and positively correlates with age, similarly to what we observed in both mouse and NMR (*r* = 0.463, *p* = 2.67E-06). In addition, a CpG site in the promoter region of human *Mab21l2* (cg05878390 in the 450k array) was among the top 20 significant differentially methylated CpGs associated with age in a fetal lung dataset of in-utero-smoke and control samples at 57–122 days of gestation^50^. This gene was also differentially methylated in the dorsolateral prefrontal cortex of opioid users in humans, and there was a significant association between opioid use and the Levine phenotypic age^51^. Thus, *Mab21l2* emerges as a conserved gene involved in the epigenetic aging process. In addition, *Mab21l2* showed higher expression in mesenchymal stem cells of elderly subjects (79–94 years old) suffering from osteoporosis, which is an age-related disease characterized by an imbalance in bone homeostasis, compared to age-matched controls^52^.

Spontaneous tumors are rare in NMRs, although most rodents, including mice, die from cancer^3^. Several genes that include NMR clock sites are associated with cancer in humans. Thus, we observed increased methylation in *Tert* promoter that may contribute to the cancer resistance of NMRs. While cancer cells maintain telomere length for unlimited growth^53^, interestingly the prolonged maintenance of the telomere length^40^ does not appear to be beneficial in tumor growth in NMR. A gene expression analysis showed that *Lrba* facilitates cancer cell growth in humans^54^, and methylation alteration of *Shank1* is predictive of chronic lymphocytic leukemia^55^. A recent study also suggested that *Neurl1b* participates in colon cancer as a tumor suppressor gene^56^. Finally, Protocadherin-7 (the gene product of *Pcdh7*) induced metastases in the bone following breast cancer^57^.

Another study revealed, based on analyses using array-based DNA methylation clocks, slower aging of NMR queens compared to other animals in the colony^31^. The study analyzed the blood samples of 18 queens with ages up to 26 years. While the association was strong (*p* = 0.00013 based on the NMR pan-tissue clock), it was less prominent (*p* = 0.031) when the two oldest queens were omitted from the analysis and considered only animals with age <15 years. Our current study examined RRBS data of 6 queens with ages up to ~12 years and observed that age acceleration of the oldest queens (>10 years old) was slightly lower compared to the younger queens (*p* = 0.043). Together, the data are consistent with the idea that NMR queens age slightly slower, and a that this effect is observed more prominently when older animals were considered. It was previously reported that breeders exhibit higher survival than non-breeders; however, there was no difference between male and female breeders^6^. Breeding males did not show difference (relative to non-breeders) in the aging rate in the cohort examined in the present study or in the cohort in another report^31^.

Overall, several lines of evidence demonstrate epigenetic aging of the NMR blood. Considering the importance of age-related epigenome remodeling and its role in the aging process itself, the data strongly suggest that these animals indeed age, even if they do not show signs of demographic aging.

## Methods

### Ethical statement

Our research complies with all relevant ethical regulations. Animal experiments were conducted in accordance with animal protocols approved by the University of Illinois Institutional Animal Care and Use Committees and the University of Rochester Committee on Animal Resources with the protocol number 2009-054.

### Animals and blood collection

Twenty of the NMRs examined in this study were maintained at the University of Illinois at Chicago, and eighty-seven NMRs were housed at the University of Rochester. NMRs were kept at a controlled temperature (30 °C) and humidity (50–60%) with access to food *ad libitum*. All animals in the Rochester colonies were microchipped. To collect blood, animals were placed headfirst into a plastic restraining cone (decapicone, Braintree Scientific) such that the tail remained accessible outside of the cone. The tail was then positioned on a benchtop, ventral side up. A sterile razor blade was used to make a superficial incision across the ventral surface of the tail about halfway between the body and end of the tail. The incision induced bleeding from the tail vein. The animal was then positioned above a blood collection tube (BD Microtainer, tubes with K2EDTA, Becton, Dickinson and Company) so that blood drops from the incision were collected into the collection tube. The collected blood was immediately snap-frozen. After the blood collection, gentle pressure was applied to the tail incision site for one to two minutes to stop further bleeding. Animals were monitored for a few days after the procedure.

### RRBS

The blood DNA was extracted by DNeasy Blood & Tissue Kit (Qiagen 69506) and cleaned up using RNase treatment followed by concentration. The DNA was eluted from Qiagen columns in 100 µl of 10 mM Tris-HCl buffer, pH 8.0. RNA removal was performed with 2 µl of RNase A (Life Technologies) at room temperature for 2 min and isolated genomic DNA was purified using Genomic DNA Clean & Concentrator™-10 (Zymo D4011), eluted in 25 µl of 10 mM Tris-HCl buffer, pH 8.0, and quantified using a Qubit 2.0 (Life Technologies AM2271). RRBS libraries were prepared from 100 ng of purified DNA per sample following a protocol described elsewhere^20^. The libraries were sequenced with Illumina HiSeq2500, PE150. In total, 20% of PhiX genomic DNA was spiked to compensate for the low complexity of the libraries.

### Analysis of sequence reads

Reads were trimmed and quality filtered by TrimGalore! v0.4.1 using the–rrbs option for RRBS. Methylation levels were extracted using Bismark v0.15.0^58^ with Bowtie 2^59^ mapping to the NMR genome (HetGla_female_1.0). As the number of contigs exceeded the maximum allowed to be used in mapping, we concatenated short contigs (<1,000,000 bp) into a pseudocontig separated by 1000 ‘N’. For further analysis, we used only the CpG sites that are covered by at least 5 reads in at least 90% of the samples.

### Shannon entropy

First, we calculated the Shannon entropy of each individual CpG site of a sample as follows:

$${{{\mbox{Entropy}}}}\left({{{{C}}{{p}}{{G}}}}_{i}\right)=-{{m}}_{i}{* {{\log }}}_{2}{m}_{i}-({1-m}_{i})* {{{\log }}}_{2}({1-m}_{i}),$$

where ${{m}}_{i}$ is the methylation level of the *i*th CpG site. Then, the average entropy of all 3,089,098 CpG sites provided the methylome entropy of the sample (mean entropy). The same entropy calculation was used for human and mouse.

### Density plot

The density plot (Fig. 1d) was calculated by kernel density estimation (KDE) for 11 representative samples, each being randomly selected from each year group (P9m6, 0.75y; G58, 1.82y; G54, 2.85y; G50, 3.29y; G15, 4.37y; G81, 5.29y; G69, 6.85y; G23, 7.83y; G47, 8.07y; G12, 10.05y; and G45, 11.63y).

### Promoter methylation

Promoter region of each gene was determined as [−1500, +500] bp from transcription start site following the direction of transcription by using the Ensembl annotation file hetGla2.ncbiRefSeq.gtf. Then, mean methylation level of CpG sites in the promoter regions were calculated for each gene and sample.

### Development of the NMR blood clock

Using the CpG methylation levels based on RRBS profiling, we generated a methylation profile table (feature table) that contains 430,080 CpG sites that were sufficiently covered (by at least 5 reads) in all 107 samples of our study. We trained an ElasticNet regression model using the random 80% of the samples (*n* = 85) and tested on the remaining samples (*n* = 22). The lambda parameter was optimized on the training set by the built-in 10-fold cross-validation of the Python package Glmnet (https://github.com/civisanalytics/python-glmnet, v2.2.1; alpha=0.5, n_splits=10). The final model referred to as the ‘NMR blood clock’ in the study. We provided the intercept and the non-zero weights (Supplementary Data 4). The clock requires the input methylation levels to be between 0 and 1 inclusive and provides predictions in years. To achieve reliable age predictions for all of the study sample, we performed a 5-fold cross-validation repeating the same procedure as above. The 5-fold cross validation resulted in 5 clocks tested in 5 independent sets covering all of the samples (Fig. 2b, Supplementary Fig. 2, Supplementary Data 3). *Clock 1* (referred to as ‘NMR blood clock’) was used for further analysis.

### Gene analysis

For gene analyses, we used the HetGla_female_1.0/hetGla2 naked mole-rat genome annotation (hetGla2.ncbiRefSeq.gtf) downloaded from the UCSC Genome Browser. For each of the 26 CpG sites of the NMR blood clock, we examined gene(s) that overlap with it, including 1500 bp flanking regions upstream and downstream. If there existed such a gene (referred to as ‘NMR blood clock site gene’), we determined the genomic feature covered by the clock CpG site (e.g. exon, intron, promoter). We defined promoter regions as the regions spanning 1500 bases upstream and 500 bases downstream of the transcription start site of the corresponding gene. We calculated Pearson correlation coefficient with age for each CpG site covered by at least 5 reads in at least 90% of the study samples.

For comparison, we searched for *gene_id-s* of the NMR blood clock site genes in the *mm10* mouse genome annotation data (mm10.ncbiRefSeq.gtf) downloaded from the UCSC Genome Browser. For a hit, we analyzed 1,500 bp flanking regions of the gene upstream and downstream. We calculated Pearson correlation coefficient with age for each CpG site covered by at least 5 reads in at least 90% of the Petkovich et al. mouse blood dataset (GSE80672). We also performed the same procedure for the Thompson et al. mouse blood dataset (GSE120132).

### Age acceleration

Age acceleration was calculated as the deviation of the predicted age of test samples from the regression line of the predicted ages of all samples. We assessed if the mean age acceleration of test samples is different from zero by applying one sample t-test.

### Statistics

Correlations were evaluated by Pearson correlation coefficient (‘*r*’) and the corresponding two-sided *p*-values using the *stats.pearsonr* function of the python package scipy v1.3.1 (stats module). *P*-values of below 0.05 were interpreted as significant. We always use two-sided *p*-value in the study. Bonferroni correction was applied whenever it is mentioned in the text (in this case *p*-value below 0.05/N was considered as significant, where *N* is the number of CpG sites). Data analysis was done by using Python 3.7.4, Pandas 0.25.1, and Numpy 1.17.2.

### Reporting summary

Further information on research design is available in the Nature Research Reporting Summary linked to this article.

## Supplementary information

Supplementary Information

Description of Additional Supplementary Files

Supplementary Dataset 1

Supplementary Dataset 2

Supplementary Dataset 3

Supplementary Dataset 4

Reporting Summary

## Data Availability

Bisulfite sequence data generated in this study were deposited in the SRA database under accession code “PRJNA742002”. Processed data (i.e. methylation levels) generated in this study were deposited in the GEO database under the accession code “GSE179039”. Other data generated in this study are provided in supplementary tables. We also used publicly available datasets for cross-species comparison (Petkovich et al. “GSE80672”; Thompson et al. “GSE120132” and Hannum et al. “GSE40279”), a publicly available genome (“HetGla_female_1.0, [https://hgdownload.soe.ucsc.edu/goldenPath/hetGla2/bigZips/hetGla2.fa.gz]”) and genome annotations for the NMR and mouse downloaded from the UCSC Genome Browser portal (“hetGla2.ncbiRefSeq.gtf [http://hgdownload.soe.ucsc.edu/goldenPath/hetGla2/bigZips/genes/hetGla2.ncbiRefSeq.gtf.gz]”, “mm10.ncbiRefSeq.gtf [https://hgdownload.soe.ucsc.edu/goldenPath/mm10/bigZips/genes/mm10.refGene.gtf.gz]”). Source data of Figs. 1, 2, and 4 is provided as a Source Data file. Source data are provided with this paper. All figures and data tables can be reproduced from the processed methylation data generated by this study (along sample metadata) and public datasets. Source data are provided with this paper.

## Source data

Source Data
