# Supplementary material for: Epigenetic aging of the demographically non-aging naked mole-rat
Source: Nat Commun. 2022 Jan 17;13:355. doi: 10.1038/s41467-022-27959-9 (PMC8763950; doi:10.1038/s41467-022-27959-9)
Supplement: Supplementary file 1 — Supplementary Information [file 41467_2022_27959_MOESM1_ESM.pdf]

Supplementary Information

Epigenetic aging of the demographically non-aging naked mole-rat

Csaba Kerepesi[1#], Margarita V. Meer[1,2#], Julia Ablaeva[3], Vince G. Amoroso[4], Sang-Goo Lee[1], Bohan Zhang[1], Maxim V. Gerashchenko[1], Alexandre Trapp[1], Sun Hee Yim[1], Ake T. Lu[5], Morgan E. Levine[2], Andrei Seluanov[3], Steve Horvath[5,6], Thomas J. Park[4], Vera Gorbunova[3], Vadim N. Gladyshev[1]*

[1] Division of Genetics, Department of Medicine, Brigham and Women's Hospital and Harvard Medical School, Boston, MA 02115, USA

[2] Department of Pathology, Yale School of Medicine, New Haven, CT 06510, USA

[3] Departments of Biology and Medicine, University of Rochester, Rochester, NY 14627, USA

[4] Laboratory of Integrative Neuroscience, Department of Biological Sciences, University of Illinois at Chicago, Chicago, IL 60607, USA

[5] Department of Human Genetics, David Geffen School of Medicine, University of California, Los Angeles, CA 90095, USA

[6] Department of Biostatistics, Fielding School of Public Health, University of California, Los Angeles, CA 90095, USA

[#] These authors contributed equally

* Correspondence: vgladyshev@rics.bwh.harvard.edu

Content:

Supplementary Figure 1

Supplementary Figure 2

Supplementary Figure 3

Supplementary Figure 4

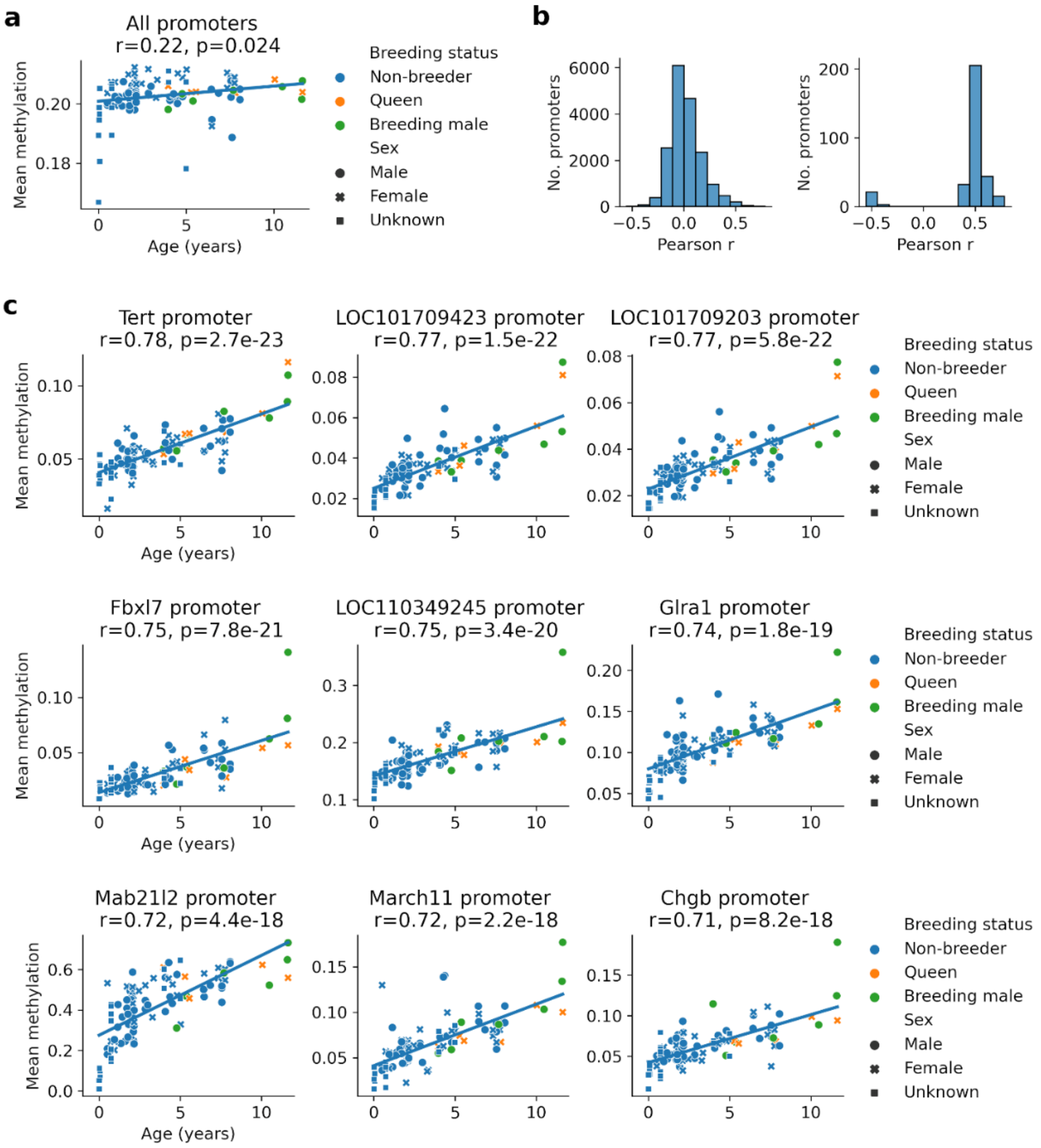

Supplementary Figure 1. Mean methylation of NMR gene promoters changes with age. a Mean promoter methylation level over all 17,823 NMR genes covered by our data. **b** Pearson correlation coefficient (*r*) of mean promoter methylation and age for each gene. Histogram of the correlation coefficients of mean promoter methylation and age for all genes (left panel) and only for the significant genes after Bonferroni correction (right panel). **c** Mean promoter methylation in the function of age for the genes that showed an *r* greater than 0.7. Pearson correlation coefficient (*r*), its two-sided p-value (*p*) without adjustment for multiple-comparison are indicated.

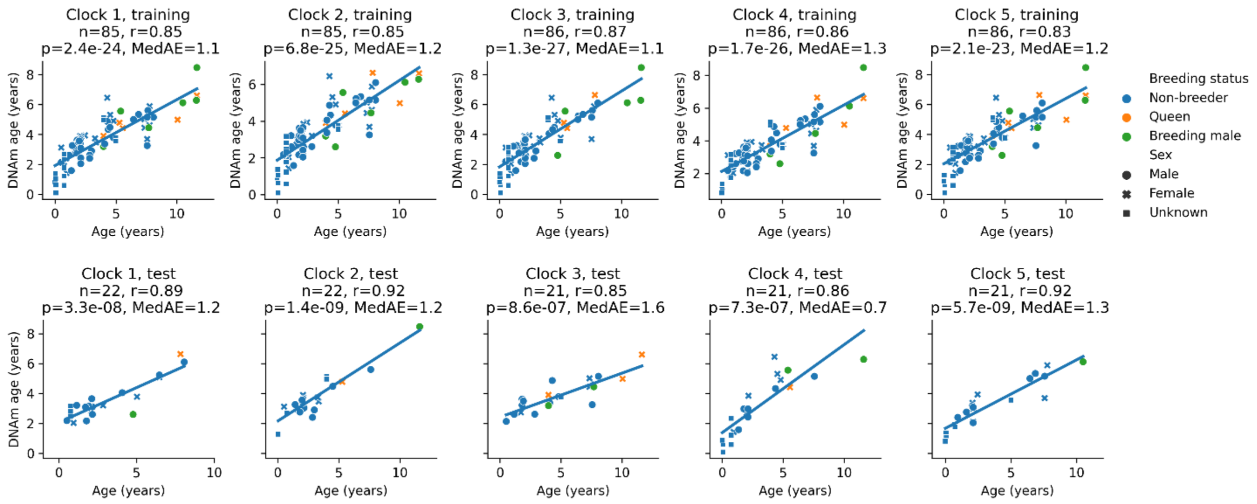

Supplementary Figure 2. Training (upper row) and test set (bottom row) metrics of five different clocks of the 5-fold cross-validation run on the 107 NMR blood samples using ElasticNet regression (Glmnet). ‘Clock 1’ is the ‘NMR blood clock’. We calculated two-sided p-values for Pearson correlation coefficients without adjustment for multiple comparison.

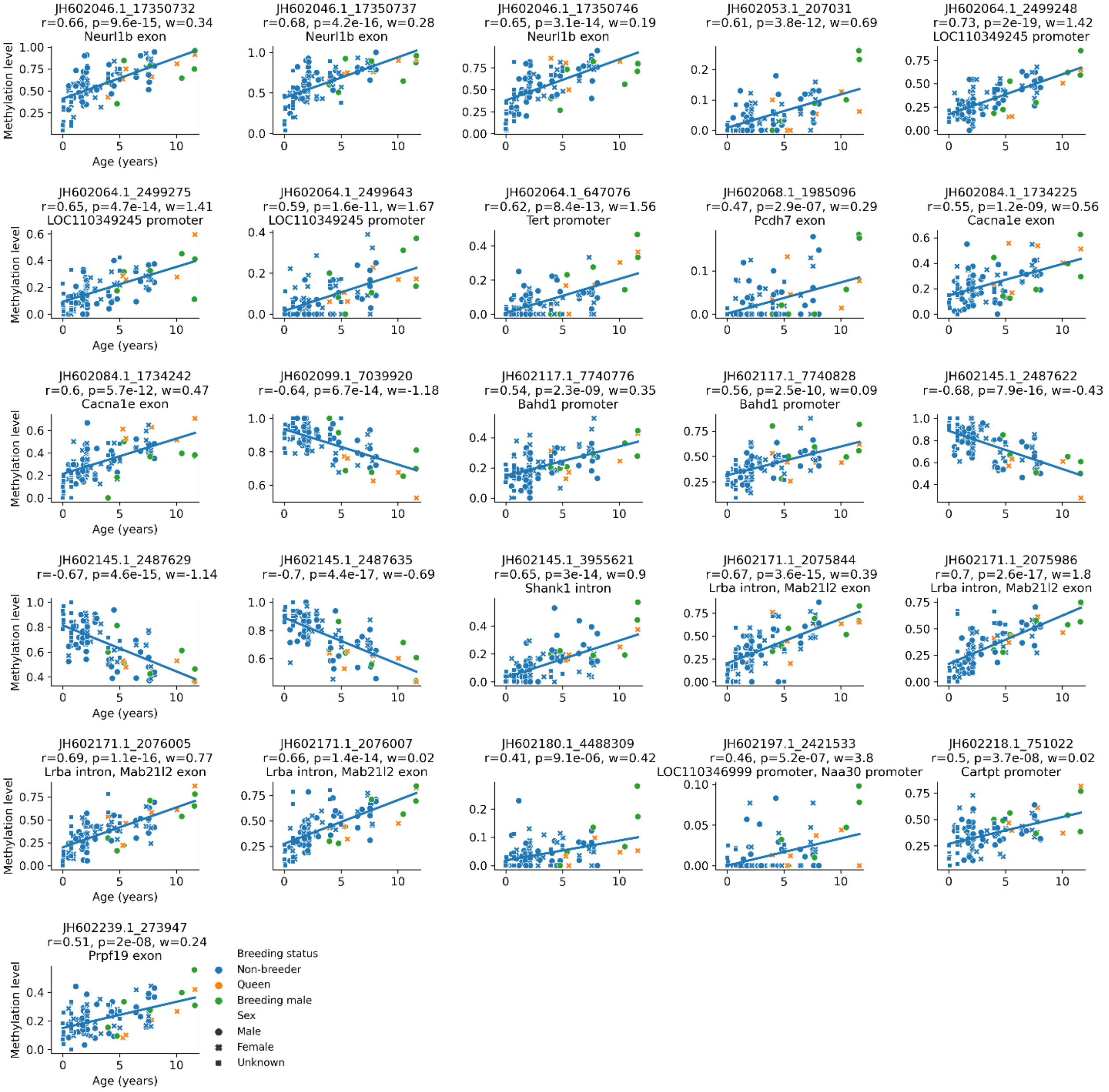

Supplementary Figure 3. **Methylation levels of the 26 CpG sites of the NMR blood clock as a function of age.** Positions of clock sites in the corresponding genomic contig are indicated on the top of the plots. Pearson correlation coefficient (*r*) and its two-sided p-value (*p*) are indicated along with the weights of the CpG sites in the NMR blood clock (*w*). We did not make adjustment for multiple comparison. Gene name and location of the CpG site in the gene sequence are also indicated (exon, intron or promoter) if the CpG site was located in a gene body or its 1,500 bp upstream or downstream flanking region. We defined promoters as regions spanning 1,500 bases upstream and 500 bases downstream of the transcription start site of the corresponding gene.

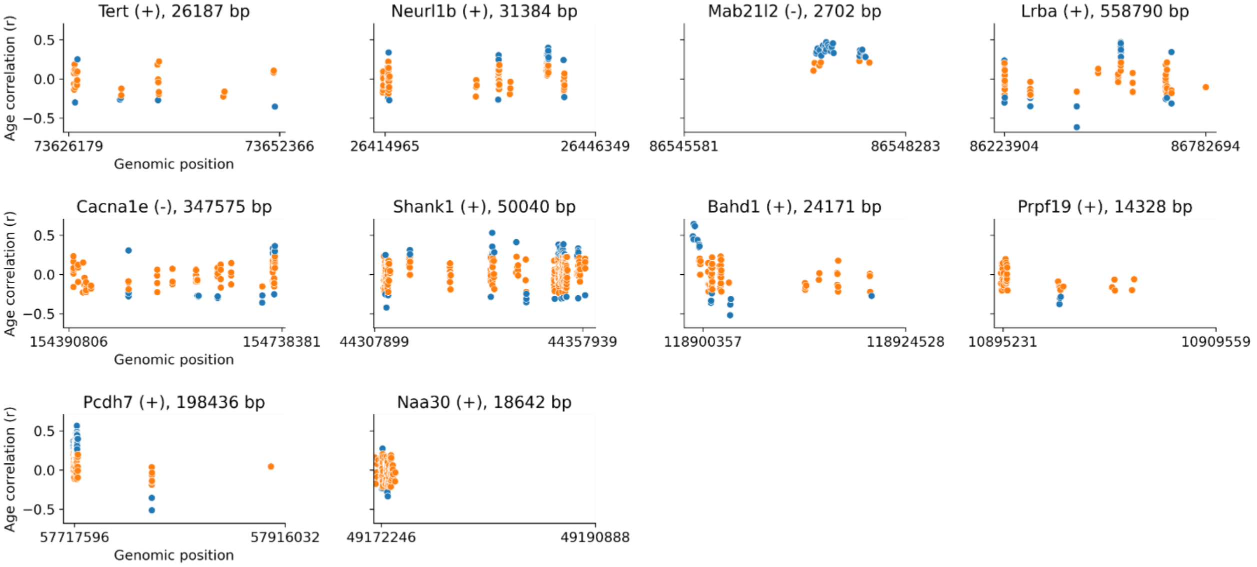

Supplementary Figure 4. Age-associated methylation patterns of mouse homologs of the NMR clock-associated genes for comparison (the second mouse blood dataset, Thompson et al.[18], for validation). Notations are the same as in Figure 3. We calculated two-sided p-values for Pearson correlation coefficients without adjustment for multiple comparison.
