# Supplementary material for: Epigenetic aging of the demographically non-aging naked mole-rat
Source: Nat Commun. 2022 Jan 17;13:355. doi: 10.1038/s41467-022-27959-9 (PMC8763950; doi:10.1038/s41467-022-27959-9)
Supplement: Supplementary file 2 — Description of Additional Supplementary Files [file 41467_2022_27959_MOESM2_ESM.docx]

**Description of Additional Supplementary Files**

File Name: Supplementary Information
Description: Supplementary Figures 1-4.

File Name: Source Data
Description: Source data for manuscript figures.

File Name: Supplementary Data 1
Description: Sample information.

File Name: Supplementary Data 2
Description: Gene promotor information.

File Name: Supplementary Data 3
Description: DNAm ages of samples.

File Name: Supplementary Data 4
Description: Weights of clock sites.
