# Supplementary material for: Epigenetic aging of the demographically non-aging naked mole-rat
Source: Nat Commun. 2022 Jan 17;13:355. doi: 10.1038/s41467-022-27959-9 (PMC8763950; doi:10.1038/s41467-022-27959-9)
Supplement: Supplementary file 7 — Reporting Summary [file 41467_2022_27959_MOESM7_ESM.pdf]

nature research

Corresponding author(s): Vadim N. Gladyshev

Last updated by author(s): Dec 20, 2021

Reporting Summary

Nature Research wishes to improve the reproducibility of the work that we publish. This form provides structure for consistency and transparency in reporting. For further information on Nature Research policies, see our Editorial Policies and the Editorial Policy Checklist.

Statistics

For all statistical analyses, confirm that the following items are present in the figure legend, table legend, main text, or Methods section.

n/a	Confirmed	
☐	☒	The exact sample size (*n*) for each experimental group/condition, given as a discrete number and unit of measurement
☐	☒	A statement on whether measurements were taken from distinct samples or whether the same sample was measured repeatedly
☐	☒	The statistical test(s) used AND whether they are one- or two-sided<br>*Only common tests should be described solely by name; describe more complex techniques in the Methods section.*
☐	☒	A description of all covariates tested
☐	☒	A description of any assumptions or corrections, such as tests of normality and adjustment for multiple comparisons
☐	☒	A full description of the statistical parameters including central tendency (e.g. means) or other basic estimates (e.g. regression coefficient) AND variation (e.g. standard deviation) or associated estimates of uncertainty (e.g. confidence intervals)
☐	☒	For null hypothesis testing, the test statistic (e.g. *F*, *t*, *r*) with confidence intervals, effect sizes, degrees of freedom and *P* value noted<br>*Give P values as exact values whenever suitable.*
☒	☐	For Bayesian analysis, information on the choice of priors and Markov chain Monte Carlo settings
☒	☐	For hierarchical and complex designs, identification of the appropriate level for tests and full reporting of outcomes
☐	☒	Estimates of effect sizes (e.g. Cohen's *d*, Pearson's *r*), indicating how they were calculated

Our web collection on statistics for biologists contains articles on many of the points above.

Software and code

Policy information about availability of computer code

Data collection	Reads generated by this study were trimmed and quality filtered by TrimGalore! v0.4.1. Methylation levels were extracted using Bismark v0.15.0 with Bowtie 2. Public data were downloaded from the GEO database or the UCSC Genome Browser website without using any specific software.
Data analysis	For the NMR clock development we used the Python package Glmnet (https://github.com/civisanalytics/python-glmnet, v2.2.1). Correlations were evaluated by Pearson correlation coefficient ('r') and the correspondent two-sided p-values using the stats.pearsonr function of the python package scipy v1.3.1 (stats module). General data analysis was done by using Python 3.7.4, Pandas 0.25.1 and Numpy 1.17.2.

For manuscripts utilizing custom algorithms or software that are central to the research but not yet described in published literature, software must be made available to editors and reviewers. We strongly encourage code deposition in a community repository (e.g. GitHub). See the Nature Research guidelines for submitting code & software for further information.

Data

Policy information about availability of data

All manuscripts must include a data availability statement. This statement should provide the following information, where applicable:

- Accession codes, unique identifiers, or web links for publicly available datasets
- A list of figures that have associated raw data
- A description of any restrictions on data availability

The bisulfite sequence data generated in this study have been deposited in the SRA database under accession code "PRJNA742002 [https://www.ncbi.nlm.nih.gov/bioproject/PRJNA742002]". The processed data (i.e. methylation levels) generated in this study have been deposited in the GEO database under accession code "GSE179039, [https://www.ncbi.nlm.nih.gov/geo/query/acc.cgi?acc=GSE179039]". Other data generated in this study are provided in the supplementary tables. Source data of Fig. 1, 2 and 4 is provided as a Source Data file. Source data are provided with this paper. We also used publicly available datasets for cross-species

comparison (Petkovich et al. “GSE80672 [https://www.ncbi.nlm.nih.gov/geo/query/acc.cgi?acc=GSE80672]”; Thompson et al. “GSE120132 [https://www.ncbi.nlm.nih.gov/geo/query/acc.cgi?acc=GSE120132]” and Hannum et al. “GSE40279 [https://www.ncbi.nlm.nih.gov/geo/query/acc.cgi?acc=GSE40279]”), a publicly available genome (“HetGla_female_1.0, [https://hgdownload.soe.ucsc.edu/goldenPath/hetGla2/bigZips/hetGla2.fa.gz]”) and genome annotations for the NMR and mouse downloaded from the UCSC Genome Browser portal (“hetGla2.ncbiRefSeq.gtf [http://hgdownload.soe.ucsc.edu/goldenPath/hetGla2/bigZips/genes/hetGla2.ncbiRefSeq.gtf.gz]”, “mm10.ncbiRefSeq.gtf [https://hgdownload.soe.ucsc.edu/goldenPath/mm10/bigZips/genes/mm10.refGene.gtf.gz]").

Field-specific reporting

Please select the one below that is the best fit for your research. If you are not sure, read the appropriate sections before making your selection.

- [x] Life sciences
- [ ] Behavioural & social sciences
- [ ] Ecological, evolutionary & environmental sciences

For a reference copy of the document with all sections, see nature.com/documents/nr-reporting-summary-flat.pdf

Life sciences study design

All studies must disclose on these points even when the disclosure is negative.

Sample size	No sample-size calculation was performed.
Data exclusions	No data were excluded from analyses.
Replication	No technical replicates were used in the study.
Randomization	Not applicable as it was not an intervention study.
Blinding	Not applicable as it was not an intervention study.

Reporting for specific materials, systems and methods

We require information from authors about some types of materials, experimental systems and methods used in many studies. Here, indicate whether each material, system or method listed is relevant to your study. If you are not sure if a list item applies to your research, read the appropriate section before selecting a response.

Materials & experimental systems

n/a	Involved in the study	
x		Antibodies
x		Eukaryotic cell lines
x		Palaeontology and archaeology
	x	Animals and other organisms
x		Human research participants
x		Clinical data
x		Dual use research of concern

Methods

n/a	Involved in the study	
x		ChIP-seq
x		Flow cytometry
x		MRI-based neuroimaging

Animals and other organisms

Policy information about studies involving animals; ARRIVE guidelines recommended for reporting animal research

Laboratory animals	107 blood samples derived from male and female naked-mole-rats (Heterocephalus glaber) ranging in age from 0.01 years to 11.63 years.
Wild animals	The study did not involve wild animals.
Field-collected samples	No field collected samples were used in the study.
Ethics oversight	Our research complies with all relevant ethical regulations. Animal experiments were conducted in accordance with animal protocols approved by the University of Illinois Institutional Animal Care and Use Committees and the University of Rochester Committee on Animal Resources with the protocol number 2009-054.

Note that full information on the approval of the study protocol must also be provided in the manuscript.
